# Ethical perceptions and uptake of sexual and reproductive health services among unmarried adolescents in Morogoro, Tanzania: a cross-sectional study

**DOI:** 10.21203/rs.3.rs-10911392/v1

**Published:** 2026-09-20

**Authors:** Baraka M. Morris, Suleiman Chombo, Connie M. Ulrich, Deodatus CV. Kakoko

**Affiliations:** Muhimbili University of Health and Allied Sciences; Muhimbili University of Health and Allied Sciences; University of Pennsylvania; Muhimbili University of Health and Allied Sciences

**Keywords:** unmarried adolescents, adolescents’ sexual and reproductive health, ethical perceptions, health service utilization, Tanzania

## Abstract

**Background:**

Tanzania has made efforts to improve access to sexual and reproductive health services (SRHS) for teenagers, yet few teens actually use them. One possible reason is that what teens expect from care may not match what their communities find acceptable. There is also limited information on whether concerns about informed consent, privacy, confidentiality, and community attitudes toward premarital sex affect whether teens seek SRHS. This study examined these concerns and their relationship to SRHS use among unmarried teens in Morogoro, Tanzania.

**Methods:**

We selected 614 unmarried teens from various areas of Morogoro. Of these, 604 agreed to participate, and 382 (63.2%) returned completed questionnaires. After excluding 70 largely incomplete ones, 312 teens were included in the final analysis. We collected information using a modified WHO questionnaire with 38 additional questions about ethical issues related to sexual and reproductive health services. We grouped similar questions to identify key ethical aspects and examined whether these aspects were linked to teens' use of the services.

**Results:**

Three main ethical aspects were identified: informed consent, moral views about premarital sex, and privacy and confidentiality. Moral views about premarital sex and privacy and confidentiality were linked to greater use of SRHS.

**Conclusion:**

Teens often feel they need their community’s approval before seeking sexual and reproductive health services, while also needing their privacy to be protected. Respecting community values while providing private, comfortable, and teen-friendly care may help improve the use of these services.

## Background

Over the past three decades, global health initiatives have increasingly prioritized adolescent-friendly healthcare, particularly sexual and reproductive health services (SRHS).^1, 2^ The 1994 International Conference on Population and Development catalyzed global commitments to advancing adolescent sexual and reproductive health and rights (ASRHR).^2^ Despite these initiatives, uptake of SRHS remains persistently low, particularly among unmarried adolescents.^3^ In many low- and middle-income countries (LMICs), especially in sub-Saharan Africa, this situation is often rooted in sociocultural norms and related ethical tensions surrounding adolescent sexuality.^1, 4^ In conservative communities, premarital sexual activity is often viewed as immoral, fostering stigma toward unmarried adolescents seeking SRHS.^5, 6^ Furthermore, requirements for parental involvement by some conservative healthcare providers may undermine adolescents’ autonomy, privacy, and confidentiality.^4, 7, 8^ Together, these structural, social, and ethical aspects highlight the need to better understand how ethical perceptions influence SRHS utilization among unmarried adolescents.

### Informed consent

is a cornerstone of respect for personal autonomy and a sentinel principle of biomedical ethics. ^9, 10^ It upholds the individual’s right to self-determination, empowering them to make decisions about their own bodies and the services they receive. This principle becomes particularly complex when applied to adolescents under 18, raising questions about their capacity to provide informed consent for care, especially for SRHS. Although adolescents often have the cognitive capacity to understand information, their judgment may not be fully mature. Consequently, ethical standards require that minors provide assent even when parental consent is obtained.^11^ The debate surrounding informed consent for adolescent SRHS is both ethical and legal, centering on the balance between self-determination and the adolescent's ability to comprehend information.^12^ In Tanzania, this debate is compounded by a contradictory legal framework: the Marriage Act of 1971 sets the legal age of consent for sexual activity at 18; yet it provides an explicit exception for married girls aged 15 or older.^13^ In practice, the age of majority for medical consent is generally 18, except for HIV testing and SRHS, for which it is 15.^14^ Across sub-Saharan Africa, research has revealed that the requirement for parental consent often poses a major barrier for unmarried adolescents, who may fear disclosing their sexual behaviors.^15, 16^ Collectively, these ethical, legal, and social factors underscore the complexity of applying informed consent principles to adolescent SRHS in practice.

Uncertainty regarding privacy and confidentiality remains a significant obstacle to the utilization of SRHS among unmarried adolescents, increasing their vulnerability to adverse sexual and reproductive health outcomes.^10, 17, 18^ Many adolescents fear that private discussions might be disclosed to parents or community members and judged negatively in accordance with familial and cultural norms. Consequently, they may avoid seeking necessary services or withhold critical information, even at the expense of their health.^9^ Assurances of privacy and confidentiality are therefore essential, as they foster trust, promote open dialogue, and facilitate timely access to care.^19^ However, these assurances are often compromised by cultural and practical realities, such as parental presence during SRH consultations, which can undermine an adolescent's sense of privacy.^16, 20^ Studies show that most adolescents would discontinue SRHS if parents were present or informed and that they are often reluctant to discuss such matters with them.^17, 21-23^ Taken together, these factors highlight an ethical and social tension between adolescents’ expectations of privacy and confidentiality and the moral norms shaping community and healthcare provider attitudes toward adolescent sexuality and SRHS utilization.

Despite national and global initiatives to promote adolescent-friendly health services in Tanzania, utilization among sexually active adolescents aged 15–19 remains low, at under 20%.^3, 18^ About one-third of health facilities in Tanzania provide youth-friendly SRH services, and the government has made efforts to improve their accessibility, acceptability, equity, and appropriateness.^3, 24, 25^ However, a disconnect remains between adolescents’ expectations, ethical standards, and community moral norms. In particular, little is known about the practical ethical aspects of informed consent, privacy, and confidentiality, which are critical determinants of SRHS utilization among unmarried adolescents. This suggests that existing services may not fully meet adolescents’ expectations, highlighting the need to examine their ethical perceptions. Accordingly, this study investigates how ethical perceptions influence SRHS uptake among unmarried adolescents in Tanzania. Specifically, it examines the roles of informed consent, moral perceptions of premarital sex, and privacy and confidentiality in service utilization.

## Material and Methods

### Study design

This cross-sectional study employed a quantitative household survey to examine ethical aspects of SRHS utilization among unmarried adolescents aged 15–19 years in Morogoro, Tanzania, as described in detail elsewhere.^26^ As previously described, data were collected for the main study from five wards representing urban (Kiwanja cha Ndege), peri-urban (Bigwa, Kihonda), and rural (Mlali, Mangae) settings within Morogoro Municipal Council and Mvomero District.^27^ These wards were identified through the multistage sampling procedure described below. This region was purposively selected because it has a persistently high teenage pregnancy rate that remains above the national average of 22%.^28-30^ Health facilities in the region also provide the national adolescent-friendly SRHS package to prevent STIs, HIV, teenage pregnancies, and gender-based violence, while promoting nutrition, education, and life skills.^3^

### Sample size and sampling

The sample size and sampling procedures were consistent with those described by Morris et al.^26^ Using Cochran’s single-proportion formula with a 5% margin of error and 95% confidence level, the initial calculated sample was 348. Applying a design effect of 1.5 for the multistage sampling design increased the required analytical sample to 522, and a further 15% allowance for non-response resulted in a final target sample size of 614 participants. A total of 605 adolescents were contacted; 604 (99.8%) consented to complete the survey. Recruitment was terminated after 605 adolescents were approached (604 consented and 1 declined) because high adolescent mobility and inaccurate household listings prevented attainment of the 614-participant target within the permitted timeframe and budget. Of the 604 who consented, 382 returned the survey, yielding a response rate of 63.2%. During data preparation, 70 returned questionnaires were excluded because they were substantially incomplete, including missing data for the outcome variable and the entire ethical aspects section. Therefore, 312 participants were included in the final analysis, representing 59.8% of the target analytical sample size of 522.

A multistage sampling strategy was employed, beginning with the random selection of two districts. Three of the 29 wards in the municipal district and two of the 27 wards in the rural district were then randomly selected. Within the selected wards, 12 streets and six villages were randomly sampled. In the absence of a household sampling frame, local field guides were used to identify households with eligible adolescents, and one eligible adolescent was enrolled per household, prioritizing the eldest when applicable. Proportionate sampling guided enrolment across the selected sites. Data collection was conducted within a single day at each site to minimize potential information sharing among participants. Adolescents were excluded if they were cohabiting or if a parent or guardian reported a history of mental illness that could affect their ability to participate meaningfully or provide informed consent or assent.

The ethical aspects module was completed by 312 participants, accounting for 51.7%, which represents 59.8% of the minimum required analytical sample of 522 participants. The lower completion rate of this module was partly attributable to the initial questionnaire administration procedure, wherein participants were permitted to retain questionnaires for up to 24 hours. This may have compromised perceptions of privacy and confidentiality, particularly when completing the culturally sensitive sexual behaviour section. Subsequently, the procedure was revised to require a two-hour completion window, with sealed envelopes provided for returning the questionnaires. Following this adjustment, module completion rates improved. The reduced size of the analytical sample may have affected the precision of the estimates and heightened the risk of non-response bias, both of which are recognized as limitations of the study.

### Data Collection

We adapted an adolescent structured questionnaire developed by John Cleland and made available from the World Health Organization (WHO) that is reported elsewhere.^26, 31^ For the purpose of this paper, our goal was to assess ethical aspects in the utilization of SRHS among adolescents by adding 38 items to the original WHO questionnaire sections (i.e., socioeconomic and family characteristics, experience of heterosexual contact, and experience of SRHS). A small pilot study was conducted on the additional ethics items with 46 participants, yielding a Cronbach’s alpha reliability coefficient of 0.74. Based on feedback from the pilot study, minor revisions were made to item wording to improve clarity and ensure socially appropriate terminology.

Data were collected between June 21, 2023, and February 2, 2024, following permission from local authorities in the Morogoro region. We obtained informed consent from parents or legal guardians and assent from adolescents under 18 years of age. Participants aged 18 years and older provided informed consent independently. Eligible participants completed the questionnaire privately and sealed it to ensure confidentiality, with guidance from research assistants where necessary. The questionnaire is available from the corresponding author upon request.

### Variables

The primary outcome variable was whether unmarried adolescents had ever utilized SRHS (yes/no). The independent variable included the newly added ethics items to the main questionnaire, with each item measured on a 6-point Likert scale ranging from 0 (not at all) to 5 (a very great extent), with higher scores reflecting. These items captured various ethical perceptions including questions that assessed adolescents’ autonomous decision-making capacity regarding SRHS, including their ability to access and comprehend relevant information and utilize services without parental involvement; perceptions on perceived approval of SRHS use by parents, communities, peers, and religious/cultural institutions, along with attitudes toward premarital sex; facility-level practices, including parental involvement requirements, confidentiality protections during provider consultations, and privacy-supporting physical and social environments. Finally, several items addressed perceived safety of parent-free SRHS access, trust in healthcare providers, equitable service provision, and gender inclusivity (e.g., boys’ accessibility). Sociodemographic covariates included age group, sex, residence, education level, and whether the adolescent lived with both parents.

### Data Analysis

Statistical analysis was conducted using STATA version 18, incorporating both descriptive and inferential statistics. During data preparation, 10 of the 38 original items were removed due to a high proportion of missing responses (> 50%). For the remaining items with ≥ 5% missing data, multiple imputation was performed because the data were missing at random (MAR). The demographic characteristics of the sample and ethical aspect items were summarized using measures of central tendency. This item refinement yielded an improved Cronbach’s alpha reliability coefficient, increasing from 0.74 to 0.82. Exploratory Factor Analysis (EFA) was conducted to identify latent constructs underlying perceived ethical aspects in the utilization of SRHS among unmarried adolescents. Principal Axis Factoring was employed because it is appropriate for identifying underlying constructs among correlated observed variables. Data suitability for factor analysis was assessed using Bartlett’s test of sphericity and the Kaiser–Meyer–Olkin (KMO) measure of sampling adequacy. A correlation matrix was used to examine inter-item relationships. Items were assigned to factors based on their highest factor loadings (≥ 0.50), with cross-loading items carefully evaluated.

Factor retention and the final structure were determined based on eigenvalues greater than one, scree plot inspection, and factor loadings. The cumulative variance was used to assess the overall explanatory power of the model, while extraction communalities were used to evaluate item-level performance.

For inferential analysis, a modified Poisson regression model with robust standard errors was employed to examine ethical aspects in the utilization of SRHS among adolescents. This modelling approach was selected as the outcome prevalence (18%) exceeded the 10% threshold for common events.^26^ Both univariable and multivariable models were fitted, with the multivariable model including factor-derived constructs from EFA and adjusting for sociodemographic characteristics to ensure model parsimony and theoretical relevance.^32^ Results were reported as crude risk ratios (RR) and adjusted risk ratios (aRR), with statistical significance defined as p < 0.05. Model fit was assessed separately for each ethical domain using binned residual plots, which indicated acceptable model performance with residuals randomly distributed within ± 2 standard deviations, thereby supporting good model fit.

### Ethical Considerations

This study received ethical clearance from the MUHAS Institutional Review Board (MUHAS-IRB; IRB No. MUHAS-REC-03-2021-521) and the National Health Research Ethics Committee (NaTHREC; Ref. No. NIMR/HQ/R.8c/Vol.1/2371), as documented in an earlier publication.^26^ The informed consent process for participants under 18 involved age-appropriate explanations and assent, followed by parental or guardian consent, while those aged 18–19 provided informed consent themselves. Given the sensitivity of SRHS information, privacy and confidentiality were ensured through private tool completion, coded identifiers, sealed envelopes, password-protected electronic files, and locked storage for paper records accessible only to the research team.

## Results

### Demographic Characteristics

Of the 604 adolescents recruited, 312 unmarried participants returned complete questionnaires, yielding a 51.7% response rate.^26^ The mean age of respondents was 17 years (SD = 1.4), with 58% younger than 18 (Table 1). The sample was predominantly female (58%), urban-based (75%), and relatively well educated, with 69% having attained secondary schooling or higher. Half lived with both parents. Notably, only 18% had ever accessed SRHS.

### Exploratory Factor Analysis (EFA)

**Figure 1:**
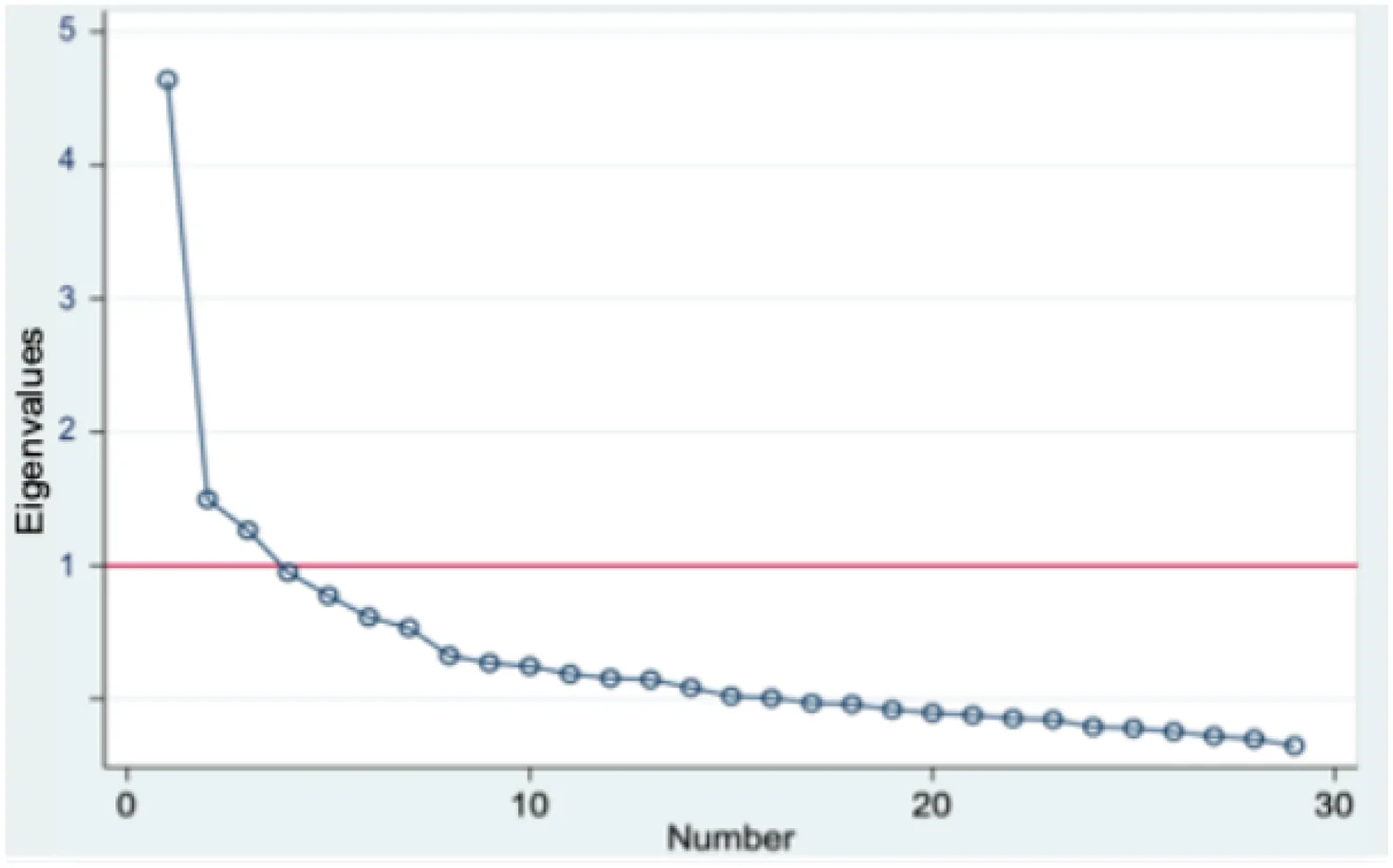
Eigenvalue scree plot suggesting three retained factors (SRHS utilization, Morogoro, 2023; N=312).

Prior to conducting the EFA, Bartlett’s test indicated that the correlation matrix differed significantly from an identity matrix (p < 0.001), confirming sufficient correlations among variables. The KMO value of 0.76 exceeded the recommended threshold of 0.50, indicating adequate sampling adequacy. Three of the four latent ethical aspects exhibited Eigenvalues > 1 (Fig. 1), were retained, and named based on factor loadings for their constituent items. Scree plot visualization supported their retention, confirming their relevance among adolescents who accessed SRHS.

#### Principal axis factor.

EFA using principal factor extraction with Promax rotation (pattern matrix) identified three factors, explaining 83.59% of the total variance before rotation (Factor 1 = 54.86%, Factor 2 = 15.88%, and Factor 3 = 12.85%). The first factor represented *moral perceptions of premarital sex and SRHS*, with strong loadings for peer SRHS use (λ = 0.6604), cultural permissiveness toward premarital sexual relationships (λ = 0.5878), and parental approval of sexual relationships (λ = 0.7241) (see Supplementary Table S1). The second factor reflected *informed consent*, with salient loadings for ability to make informed SRHS decisions (λ = 0.5213), understanding SRHS information (λ = 0.5904), and cultural support for SRHS use (λ = 0.5756). The third factor captured perceptions *of privacy and confidentiality*, with loadings for adolescent social interaction opportunities (λ = 0.5546) and facility design promoting privacy and confidentiality (λ = 0.7330). Among items with factor loadings ≥ 0.50, extraction communalities ranged from 0.21 to 0.47, with most items showing moderate representation (> 0.30). The fourth domain of the ethics items, Safety and Fairness, did not yield any item with loadings ≥ 0.50.

### Associations Between Ethical Perceptions and SRHS Uptake: Poisson Regression Findings

Modified Poisson regression analysis was conducted to examine the associations between informed consent perceptions, moral perceptions of premarital sex and SRHS, and privacy and confidentiality, and SRHS utilization among unmarried adolescents. The informed consent domain was analyzed separately due to high collinearity with the other ethical domains. After adjusting for age, sex, residence, and education level, informed consent perceptions were not significantly associated with SRHS utilization (Table 2).

A separate modified Poisson regression analysis examined the association between moral perceptions of premarital sex and SRHS and service utilization. After adjusting for the same sociodemographic covariates, moral perceptions remained significantly associated with SRHS utilization (aRR = 1.34, p = 0.025; Table 3).

### Moral perceptions = Moral perceptions of premarital sex and SRHS

After adjusting for the same sociodemographic covariates, privacy and confidentiality perceptions were significantly associated with SRHS utilization (aRR = 1.48, p = 0.013; Table 4

## Discussion

This study builds upon our previous publication, which reported that SRHS utilization among unmarried adolescents in Tanzania was low. ^26^ This level of uptake remains substantially below the One Plan III (2021–2026) target of increasing utilization from 30% to 80%.^18^ The present findings advance this work by demonstrating that ethical domains are significantly associated with service use. Specifically, adolescents’ moral perceptions of premarital sex and SRHS, together with concerns regarding privacy and confidentiality, were significantly associated with SRHS uptake.

Consistent with existing literature, this study demonstrates that privacy and confidentiality are central determinants of adolescents’ uptake of SRHS, particularly within culturally sensitive care settings.^33^ Adolescents are more likely to utilize SRHS when assured that their personal information and service use will remain private and confidential. Conversely, fears of parental discovery, unsupportive facility environments, and concerns about breaches of confidentiality discourage care seeking.^34-36^ In routine service settings, these concerns manifest through long waiting times, shared waiting spaces with adults, discomfort among boys when accessing services alongside women, and uncertainty regarding providers’ ability to consistently uphold confidentiality.^37^ Even in facilities where confidentiality is articulated as a core value, inconsistencies in implementation continue to undermine adolescent engagement with SRHS.^33, 38^ These findings underscore the need to reassess infrastructure, service delivery models, and facility routines to ensure privacy and confidentiality are treated not merely as service values, but as essential ethical requirements for improving SRHS uptake among unmarried adolescents. Future research should systematically explore how adolescents themselves define privacy and confidentiality, and the assurances they require, in order to strengthen service delivery and enhance SRHS uptake.

This study also demonstrated a significant association between adolescents’ moral perceptions of premarital sex and sexual and reproductive health services (SRHS) and their uptake of these services. Specifically, the study examined unmarried adolescents’ perceptions of parental, religious, and community approval of premarital sex and SRHS in relation to service utilization. In contexts where premarital sexual activity is socially and morally constructed as unacceptable and taboo, seeking SRHS may be perceived as evidence of sexual activity, thereby exposing adolescents to anticipated stigma and moral scrutiny.^39, 40^ Despite these prevailing norms, evidence indicates that a substantial proportion of adolescents are sexually active^3, 28^, creating a tension between normative expectations and adolescents' lived sexual realities. This dissonance can generate internalized guilt and fear of being perceived as sexually immoral, thereby reducing adolescents’ willingness to use SRHS and services that support their well-being.^37, 41^ Furthermore, parents are recognized as central moral agents in shaping adolescents’ values and behaviours; however, many may lack the knowledge, skills, or confidence to provide effective guidance on sexual behaviour and morality.^42, 43^ Consequently, adolescents often rely on alternative sources, such as peers and media, which may offer misleading or incomplete information regarding the sexual moral expectations of their community.^44^ Collectively, these findings underscore the interplay between moral perceptions, social norms, and parental guidance in shaping adolescents’ uptake of SRHS. They highlight the need for culturally responsive SRHS delivery that acknowledges prevailing moral contexts while addressing adolescents’ lived sexual realities and evolving moral understanding. Moreover, these results call for further research to examine how community-level moral norms can be constructively engaged to foster greater acceptance of SRHS packages, with the ultimate aim of improving service utilization.

### Limitations

Several limitations are noted. First, although the sample size was smaller than expected and based on 312 participants, the confidence intervals remained at 95%. However, this may have reduced the precision of estimates and introduced non-response bias. Future studies could consider online or digital data collection with completion of key modules before submission to improve module completion and enhance estimate precision. Adolescents who live in rural areas in Tanzania might not have access to online resources, so our initial approach was an appropriate method for obtaining information for the research questions. Second, the ethical items added to the WHO adolescent survey require further psychometric evaluation; however, the acceptable internal consistency observed in the present study provides preliminary evidence of their reliability in this context. Although assumptions for exploratory factor analysis (EFA) were met, one of the four initially extracted factors was excluded because of low factor loadings, potentially omitting a relevant dimension, particularly given the importance of safety in adolescent SRHS. In addition, using factor scores as observed predictors in the modified Poisson regression did not account for item-level measurement error. Finally, the cross-sectional design and potential wording and response biases limit causal inference and generalizability. Future research should incorporate qualitative approaches to explore adolescents’ concerns regarding privacy and confidentiality and examine perceptions of SRHS among parents, religious leaders, and community members in Tanzania.

### Conclusion and Recommendations

This study provides quantitative evidence that ethical perceptions related to premarital sex, as well as privacy and confidentiality, significantly contribute to unmarried adolescents' uptake of sexual and reproductive health services in Morogoro, Tanzania. It highlights that adolescents often need perceived community moral approval to seek SRHS, yet their need for privacy and confidentiality remains critical. Assurance of privacy and confidentiality was positively associated with service utilization, and adolescents were more likely to use SRHS when they perceived community approval of premarital sexual activity and service provision. Conversely, moral taboos stigmatizing premarital sex deter unmarried adolescents from seeking SRHS while increasing ethical sensitivity and the need for strong privacy and confidentiality safeguards. These findings demonstrate that ethical perceptions are not merely correlates but key contributors to service uptake and should be considered foundational requirements for youth-friendly SRHS delivery. Navigating the tension between community moral expectations and adolescents’ need for confidential and youth-friendly SRHS requires integrating these ethical considerations into service design and implementation to improve uptake.

## Supplementary Material

This is a list of supplementary files associated with this preprint. Click to download.

• TableS1EFAItemFactorLoadingandComunalityExtractionResults.docx

## Figures and Tables

**Table 1:** Sociodemographic and Sexual Characteristics of 312 Unmarried Adolescents and Their SRHS Utilization in Morogoro, Tanzania

Variables	Category	N (%)
Age Groups (Years)	Under 18	182 (58.3)
	18–19	130 (41.7)
Gender	Female	180 (57.7)
	Male	132 (42.3)
Residence	Urban	233 (74.7)
	Rural	79 (25.3)
Education Level	Primary	97 (31.1)
	Secondary/College	215 (68.9)
Ever used SRHS	No	**256 (82.1)**
	Yes	**56 (17.9)**

**Table 2:** Modified Poisson Regression of the Association Between Informed Consent and SRHSUse, Morogoro, Tanzania (N = 312)

Variable	RR	95% CI	P-Value	aRR	95% CI	P-Value
Age (Years)	1.15	0.94–1.39	0.165	1.16	0.95–1.41	0.135
Sex						
Fermale (Ref.)	1.00	_-_	0.615	1.00	_-_	0.527
Male	0.88	0.54–1.44		0.85	0.53–1.39	
Residence						
Urban (Ref.)	1.00	_-_	0.534	1.00	_-_	0.468
Rural	1.18	0.70–1.98		1.21	0.72–2.01	
Education Level						
Primary (Ref.)	1.00	_-_	0.851	1.00	_-_	0.530
Secondary/College	0.95	0.57–1.58		0.85	0.51–1.41	
Informed Consent	1.15	0.91–1.45	0.237	1.17	0.92–1.49	0.195

**Table 3:** Modified Poisson Regression of the Association Between Moral Perceptions and SRHSUse, Morogoro, Tanzania (N = 312)

Variable	RR	95% CI	P-Value	aRR	95% CI	P-Value
Age (Years)	1.15	0.94–1.39	0.165	1.14	0.94–1.39	0.182
Sex						
Fermale (Ref.)	1.00	_-_	0.615	1.00	_-_	0.489
Male	0.88	0.54–1.44		0.84	0.52–1.36	
Residence						
Urban (Ref.)	1.00	_-_	0.534	1.00	_-_	0.445
Rural	1.18	0.70–1.98		1.22	0.73–2.02	
Education Level						
Primary (Ref.)	1.00	_-_	0.851	1.00	_-_	0.776
Secondary/College	0.95	0.57–1.58		0.93	0.56–1.53	
Moral Perceptions	1.35	1.03–1.75	**0.027**	1.34	1.04–1.74	**0.025**

**Table 4:** Modified Poisson Regression of the Privacy and Confidentiality and SRHS Use, Morogoro,Tanzania (N = 312)

Variable	RR	95% CI	P-Value	aRR	95% CI	P-Value
Age (Years)	1.15	0.94–1.39	0.165	1.11	0.92–1.35	0.279
Sex						
Female (Ref.)	1.00	_-_	0.615	1.00	_-_	0.583
Male	0.88	0.54–1.44		0.87	0.54–1.42	
Residence						
Urban (Ref.)	1.00	_-_	0.534	1.00	_-_	0.391
Rural	1.18	0.70–1.98		1.25	0.75–2.06	
Education Level						
Primary (Ref.)	1.00	_-_	0.851	1.00	_-_	0.505
Secondary/College	0.95	0.57–1.58		0.84	0.51–1.39	
Privacy and Confidentiality	1.49	1.09– 2.04	**0.012**	1.48	1.09–2.02	**0.013**

## Abbreviations

ASRHR: Adolescent Sexual and Reproductive Health and Rights
EFA: Exploratory Factor Analysis
HIV: Human Immunodeficiency Virus
IRB: Institutional Board Review
LMICs: Low and Middle-Income Countries
MUHAS: Muhimbili University of Health and Allied Sciencce
NaTHREC: National Health Research Ethics Committee
NIMR: National Institution of Medical Research
SRHS: Sexual and Reproductive Health Services
WHO: World Health Organization
